# The simplified modification of diet in renal disease equation as a predictor of renal function after coronary artery bypass graft surgery

**Published:** 2010-02

**Authors:** Marius J Swart, Arlette M Bekker, Jannes J Malan, Anton Meiring, Zorada Swart, Gina Joubert

**Affiliations:** Bloemfontein Medi-Clinic, Bloemfontein, South Africa; Medical student, School of Medicine, University of the Free State, Bloemfontein, South Africa; Medical student, School of Medicine, University of the Free State, Bloemfontein, South Africa; Medical student, School of Medicine, University of the Free State, Bloemfontein, South Africa; Medical student, School of Medicine, University of the Free State, Bloemfontein, South Africa; Department Biostatistics, Faculty Health Sciences, University of the Free State, Bloemfontein, South Africa

**Keywords:** CABG, kidney, renal function

## Abstract

**Background:**

After open-heart surgery, a percentage of patients have impaired renal function. This deterioration is even seen in patients with serum creatinine (s-creatinine) values that fall within the normal laboratory range, therefore s-creatinine is not an accurate reflection of renal function. Glomerular filtration rate (GFR) is a better indication of renal status. GFR can be calculated with the simplified modification of diet in renal disease (MDRD) equation – a formula that takes age, gender, race and s-creatinine level into account. The purpose of this study was to investigate the relationship between estimated GFR pre-operatively and renal impairment postoperatively.

**Methods:**

All patients who had an isolated coronary artery bypass graft (CABG) done by one surgeon in one hospital between January 2005 and October 2007 had their s-creatinine levels determined pre-operatively. Using a computer desktop calculator, the patient’s age, gender and race were used together with the s-creatinine value to estimate the GFR. Prior to CABG, all patients were grouped into the five stages of chronic kidney disease. Renal outcome postoperatively was compared with the estimated pre-operative GFR.

**Results:**

Nineteen per cent of the 451 patients had chronic kidney disease pre-operatively, as defined by the National Kidney Foundation, according to their estimated GFR. Twenty-three per cent of these patients had renal impairment after surgery. Of the patients with reasonable renal function pre-operatively only 4% had further deterioration of renal function. Mortality did not differ significantly, but patients with postoperative renal impairment stayed in hospital on average 2.4 days longer than those who had no renal impairment postoperatively.

**Conclusions:**

Patients with chronic kidney disease before CABG have a six times greater chance of developing further renal impairment postoperatively than those with reasonable renal function beforehand. There is therefore a significant relationship between estimated GFR before CABG and deterioration of kidney function after surgery. The GFR, as calculated with the simplified MDRD, is a predictor of the risk of having renal dysfunction after CABG.

## Summary

Organ dysfunction after open-heart surgery impairs the rehabilitation of these patients. The establishment of co-morbidities is therefore part of the pre-operative workup of patients for surgery. Special investigations have cost implications, but knowing the risk factors beforehand makes interventions more timely and treatment more accurate. Renal dysfunction in particular has an influence on mortality and morbidity.^1^ Kidneys are subject to damage due to heart failure, emergency surgery, intra-aortic balloon pump use, low haematocrit during cardio-pulmonary bypass (CPB), and low cardiac output. The systemic inflammatory process associated with CPB itself negatively influences kidney performance.

Serum (s) creatinine is no longer considered an accurate test to evaluate renal function. An elderly woman with little muscle mass may have impaired renal function although her s-creatinine levels may fall within the laboratory’s normal range.^2^ Glomerular filtration rate (GFR) is now considered the most accurate way of establishing renal function. However, to determine the clearance of an exogenous marker is expensive and time consuming. Even determining creatinine clearance from a 24-hour urine sample is not always practical before a cardiac operation.

An alternative to the inaccurate s-creatinine test, and the problems associated with urine creatinine clearance, or costly radio-isotope determination of GFR is the prediction of GFR from simple and readily available values. As far back as 1976, Cockcroft and Gault suggested a method to estimate the GFR based on the patient’s age, gender, body mass and s-creatinine value.^3^ More recently, the GFR is calculated from age, gender, race, and s-creatinine, s-urea and s-albumin values. This is the calculation suggested by the Modification of Diet in Renal Disease (MDRD) study group. It is considered more accurate than the method by Cockcroft and Gault and is even more precise than creatinine clearance rate.^4^ The simplified MDRD (sMDRD) equation uses only age, gender, race and s-creatinine value. The accuracy compares favourably with the standard MDRD.^5^ Estimated GFR from the sMDRD can easily be given with an s-creatinine request from the laboratory.

The application of calculated GFR has been noted in the cardiac surgical literature.^6^ A strong correlation was found between the varying degrees of renal dysfunction pre-operatively and adverse events after cardiac surgery. The purpose of our study was to investigate the association between calculated GFR pre-operatively and renal impairment postoperatively in a local setting. In the literature, the Cockcroft and Gault formula has been used more widely, but for this study, the sMDRD method was applied. This gave us an opportunity to simultaneously establish the prevalence of renal impairment in a local population with so-called normal kidney function according to s-creatinine levels.

## Methods

The target population included all patients who had a coronary artery bypass graft operation done by one surgeon in one hospital between January 2005 and October 2007. All patients had their operations performed on cardiopulmonary bypass with non-pulsatile flow at moderate systemic temperature and with cardioplegic cardiac arrest. Patients who were on renal dialysis beforehand, off-pump bypass surgery (OPCAB) cases, CABG cases with an additional cardiac procedure, and those who died intra-operatively were excluded.

S-creatinine levels were determined automatically with the Jaffe method in a Synchron LX® system with the normal range between 80 and 130 μmol/l. The patient’s age, gender and s-creatinine value were used with a computer desktop calculator to determine the GFR according to the formula for sMDRD:^7^

GFR (ml/min/1.73m^2^) = 186 × (S-Cr)^1.154^ × age^0.203^ × 0.742 (if female) × 1.21 (for black race group)

Pre-operative patients were grouped into the five stages of chronic kidney disease (CKD) as defined by the National Kidney Foundation (NKF)^8^ (Table 1). For purposes of this study, postoperative renal impairment was defined as a 50% rise in the pre-operative s-creatinine level, with or without renal dialysis.^9^

**Table 1.** Stages Of Chronic Kidney Disease

*CKD stage*	*Description*	*GFR (ml/min/1.73 m^2^)*
1	Normal	≥ 90
2	Mild ↓ in GFR	60–89
3	Moderate ↓ in GFR	30–59
4	Severe ↓ in GFR	15–29
5	Kidney failure	< 15

GFR = glomerular filtration rate.

Other information obtained from patient records included the EuroSCORE, presence of diabetes mellitus, left ventricular ejection fraction (LVEF) less than 40%, mediastinal drainage, usage of homologous blood, length of hospital stay (LOS) and mortality. The data were analysed by the Department of Biostatics at the University of the Free State. Numerical data are expressed as means. Categorical variables are indicated in percentages. Possible differences in percentages were calculated. The association between stage of renal impairment before and after the operation was calculated as a relative risk with 95% confidence interval.

This study was approved by the Ethics Committee of the Faculty of Health Sciences at the University of the Free State, Bloemfontein, and the Board of the Bloemfontein Medi-Clinic Hospital.

## Results

After the exclusion criteria were applied, 451 patients were available for the study. The mean age was 60.5 years with a male:female gender distribution of 339:112 (75.2:24.8%). Of these patients, 98 (21.7%) had diabetes mellitus. Twenty-one patients (4.7%) had a left ventricular function of less than 40% ejection fraction. As a group, the additive EuroSCORE for operative risk for mortality was 3.26%. The mean estimated GFR using the sMDRD was 74.7 ml/min/1.73m^2^. The patients were categorised into the five stages of chronic kidney disease (CKD). None fell into stage 5; the majority of patients (283 or 62.7%) had stage 2 kidney disease (Table 2).

**Table 2.** Chronic Kidney Disease According To GFR Based On sMDRD

*CKD stage*	*GFR (ml/min/1.73 m^2^)*	*Patients n (%)*
1	> 90	82 (18.2)
2	60–89	283 (62.7)
3	30–59	83 (18.4)
4	15–29	3 (0.7)
5	< 15	0 (0.0)

Table 3 shows the outcome of patients based on their pre-operative renal function. Acceptable kidney function was found in 365 patients (80.9%), but 86 (19.1%) had, per definition, chronic kidney disease (stages 3 and 4) prior to CABG. These patients with chronic kidney disease were older and had a higher operative risk with a mean EuroSCORE of 4.7 vs 2.9% for those with reasonable kidney function. The mediastinal blood losses measured over 48 hours were almost the same (mean 691 ml for those with CKD vs 700 ml in those with reasonable kidney function), but the necessity for homologous blood was higher in the impaired group (mean 1.1 vs 0.5 units per patient). The healthier patients stayed in hospital on average one day less than those with kidney failure. The mortality for the two groups was five patients (1.4%) with normal kidneys before the operation and two (2.3%) for those with prior CKD. This difference did not reach statistical significance.

**Table 3.** Kidney Function Pre-Operatively

	*GFR > 60 ml/min/1.73 m^2^ (%)*	*GFR < 60 ml/min/1.73 m^2^ (%)*	p
Patients	365 (80.9)	86 (19.1)	
Mean age (years)	59.3	65.6	< 0.0001
Mean EuroSCORE	2.9	4.7	< 0.0001
Mean mediastinal loss (ml)	700	691	0.9117
Mean homologous blood (units/patient)	0.5	1.1	< 0.0001
Post-op ↓ kidney function	14 (3.8)	20 (23.3)	< 0.0001
Mean LOS days (days)	5.6	6.8	< 0.0001
Mortality	5 (1.4)	2 (2.3)	0.6229

GFR = glomerular filtration rate; LOS = length of hospital stay.

After the operation the patients were again classed in two groups; 417 patients (92.5%) did not have a 50% or more increase in their baseline s-creatinine levels, whereas 34 patients (7.5%) had more than a 50% increase in baseline s-creatinine (Table 4). The two groups differed in terms of age and EuroSCORE risk. Those who had renal impairment postoperatively had a lower pre-operative GFR (mean 55.8 ml/min/1.73 m^2^) compared to those without any further renal impairment (mean 76.1 ml/min/1.73m^2^). Eleven (11.2%) patients with diabetes mellitus had a deterioration of kidney function postoperatively, and 23 (6.5%) without diabetes had significantly increased s-creatinine levels. However, this difference did not reach statistical significance.

**Table 4.** Kidney Function Postoperatively Based On S-Creatinine Change From Baseline

	*< 50% rise in Cr n (%)*	*> 50% rise in Cr n (%)*	p
Patients	417 (92.5)	34 (7.5)	
Mean age (years)	59.8	68.8	< 0.0001
Mean EuroSCORE	3.1	5.1	0.0002
Mean sMDRD (ml/min/1.73m^2^)	76.1	55.8	< 0.0001
DM (*n* = 98)	87 (88.8)	11 (11.2)	0.1183
Non-DM (*n* = 353)	330 (93.5)	23 (6.5)	
LVEF < 40% (*n* = 21)	16 (76.2)	5 (23.8)	0.0153
LVEF > 40% (*n* = 430)	401 (93.3)	29 (6.7)	
Mean LOS (days)	5.7	8.1	< 0.0001
Mortality	5 (1.2)	2 (5.9)	0.0912

Cr = creatinine; sMDRD = simplified modification of diet in renal disease; DM = diabetes mellitus; LVEF = left ventricular ejection fraction, LOS = length of hospital stay.

Left ventricular function did make a difference. Five patients (23.8%) with a left ventricular function of less than 40% had renal impairment after the operation, compared to only 29 patients (6.5%) with normal left ventricular function. The length of hospital stay was longer in those patients with significantly increased baseline s-creatinine levels (mean 8.1 vs 5.7 days). The mortality was five (1.2%) patients among those who maintained their pre-operative renal function and two (5.9%) who had further deterioration. This difference was not statistically significant.

Table 5 summarises the renal outcome of patients as per stage of CKD. Of the 86 patients who were in stages 3 and 4, 20 (23.2%) had at least a 50% increase in s-creatinine levels postoperatively, compared to only 14 (3.8%) of the 365 patients who were in stages 1 and 2 (relative risk 6.1; 95% CI: 3.2–11.5. One patient with stage 1 renal function had deterioration of his kidney function. This individual had a serious surgical bleed with hypotension and a serum haemoglobin of 2 g% soon after arrival in the intensive care unit, but fortunately he recovered.

**Table 5.** Renal Impairment According To Pre-Operative GFR

*CKD stage pre-op*	*Patients*	*Post-op impairment, n (%)*	
1	82	1 (1.2)	} 14 (3.8)
2	283	13 (4.6)	
3	83	18 (21.7)	} 20 (23.2)
4	3	2 (66.7)	
5	0		

CKD = chronic kidney disease.

Of the 34 patients with renal impairment postoperatively, three required renal dialysis to correct fluid and electrolyte imbalances. All three of these patients had s-creatinine values within the laboratory’s normal range, but with GFR of 43, 46 and 79 ml/min/1.73m^2^, respectively. This last patient who required dialysis in spite of near-normal pre-operative renal function had a failed percutanous intervention procedure and was taken to theatre as an emergency case on an intra-aortic balloon pump. He was HIV positive due to blood transfusions in the past and also had raised liver enzymes pre-operatively.

## Discussion

Not only is postoperative renal impairment considered a serious risk factor for adverse effects after CABG, but kidney disease in itself is a risk factor for coronary artery disease. In a 10-year study conducted among a group of healthy people from the general Belgian population, even mild reduction in GFR was associated with death from coronary artery disease.^10^ These patients were exposed to other cardiovascular risk factors typically associated with coronary artery disease.^11^ It is now advisable that s-creatinine determination as a means of assessing renal function is no longer acceptable and in fact measurement of creatinine clearance using 24-hour urine samples does not provide a more accurate GFR than do prediction equations. These two facts are considered level A recommendations.^12^

In the present study, renal function was determined with an estimated GFR based on the sMDRD equation. The Cockcroft and Gault equation has been used more commonly to address the association between pre-operative renal function and post CABG outcome.^13^,^14^ The sMDRD uses only age, gender and s-creatinine level to calculate an estimated GFR.

All the patients were grouped according to the NKF stages for kidney function. Only 18.2% had normal kidney function (stage 1). A GFR of 60 ml/min/1.73m^2^ was used as cut-off point and 19.1% of patients had chronic renal impairment pre-operatively. This was surprisingly high as only 11 patients had s-creatinine values higher than 141 μmol/l. The patients with impaired renal function were older, however the sMDRD takes age into account. The EuroSCORE uses s-creatinine levels above 200 μmol/l as a mortality risk factor and in this series only two patients fell into this category, yet the impaired group had a higher risk for mortality. The higher age would probably explain this; they were on average six years older, with every five years over 60 years contributing 1% to the risk score.

Interestingly, the postoperative mediastinal drainage did not differ much, but the impaired group required more homologous blood. One would like to believe that kidney dysfunction contributed to that, but pre-operative haematocrit values were not part of the study. We could not demonstrate a difference in mortality rate although this was clear from a large database of patients where it was demonstrated that operative mortality rate rose inversely with declining renal function.^15^ The two patients who died among those with pre-operative stages 3 and 4 CKD were the same two who had postoperative deterioration of baseline s-creatinine levels. Impaired renal function with further deterioration could therefore be a risk for mortality. However this hypothesis was not tested, as the number of patients was too small.

After the operation, the patients’ renal function were re-assessed, but this time in terms of a 50% rise in baseline s-creatinine level (Table 4). Again, those patients with impaired renal function were on average nine years older and their EuroSCORE was higher. These patients’ estimated GFR was 26.6% lower than the group with no further impairment (55.8 vs 76.0 ml/min/1.73m^2^, respectively). It was disappointing not to be able to demonstrate an influence from diabetes mellitus on postoperative kidney dysfunction. Patients with left ventricular function of less that 40% did have a higher chance of kidney dysfunction after CABG. Patients with postoperative renal dysfunction stayed in hospital on average two and a half days longer than those with no dysfunction. In the series of Antunes, renal dysfunction after surgery increased hospital stay by 3.4 days.^16^ However, he defined postoperative impairment slightly differently from us.

An important limitation was the size of the study population. When the incidence of adverse effects or complications is low, a study needs a large number of patients to be sufficiently powered. We would have expected a difference in renal outcome between diabetics and non-diabetics in terms of postoperative renal impairment. The power of this study was perhaps just too small to have demonstrated a statistical difference between 11.2 and 6.5% (Table 4). The same applies to mortality between those who developed renal deterioration and those with unchanged kidney function. Values of 1.2 and 5.9% (Table 4) were not sufficiently different for the small number of patients in this study. However, study populations of a few thousand are almost impossible in one-man practices at a single institution.^17^ Other data missing in this study, such as haematocrit, incidence of hypertension and more detailed statistical analysis could perhaps clarify the specific influence of GFR on the outcome after CABG.

## Conclusion

In this group of patients who had isolated CABG, a fifth of them had moderate to severe chronic kidney disease pre-operatively, based on their GFR as estimated by the sMDRD. This confirms the high prevalence of kidney co-morbidity in CABG patients. Another one-fifth of the patients had stage 1 renal function pre-operatively. Of the patients with chronic kidney disease, a quarter developed renal impairment after the operation. They had a six-times higher chance to develop renal impairment than those with no or mild renal dysfunction before the operation. This postoperative deterioration of kidney function caused them to stay in hospital on average two and a half days longer than the rest of the patients. We conclude that GFR as estimated with the sMDRD is a good predictor of renal impairment after CABG.
